# Practical Remarks on Lacerations of the Uterus and Vagina, with Cases

**Published:** 1826-01

**Authors:** 


*Practical Remarks on Lacerations of the Uterus and Vagina, with Cases.* By Thomas M'Keever, M.D. late Assistant to the Dublin Lying-in-Hospital. 8vo. pp. 82. London, 1824.

An interesting, though a somewhat melancholy brochure might be made of the "miseries of a medical life," among the items of which, the accident that forms the subject of Dr. M'Keever's little work, would not be the least striking. A lady of rank or influence gets married, and a host of candidates contend for the honour of assisting Dame Juno on the happy day or night that shall make the wife a mother. One, more fortunate than the rest, though not perhaps more meritorious, is selected for the office of high priest to Lucina. Labour comes on, and every thing is proceeding prosperously, but just when one more pain is expected to complete delivery, the rupture of a few muscular fibres converts the house of joy into the house of mourning, while the feelings of the fortunate candidate are only to be envied by him who is receiving clerical consolation at the foot of the gallows!

Or the haven of hope may be still more nearly gained, when chilling disappointment intervenes. Delivery may be completed, and every thing apparently safe, when a few patulous vessels unseen, are sapping the foundation of life, and all at once the unsuspecting mother becomes blanched and moribund. Such a scene has, in our own days, been too much for the nerves of philosophy, and led to the farther misery of suicide!

Rupture of the uterus is the worst of all calamities attendant on parturition. In cases of hæmorrhage, convulsions, and other untoward accidents, the practitioner frequently has it in his power, by judicious and well regulated efforts, to rescue the parent as well as her offspring from impending danger; when the uterus gives way, both lives are almost uniformly sacrificed.

Dr. M'Keever thinks there is reason to suppose that this dreadful accident occurs much more frequently among the lower than the higher classes of female society. In the Lying-in-Hospital of Dublin, in the year 1819-20 and 21, there occurred no less than twenty cases of rupture of the uterus, out of 8600 patients, or about 1 in 430. Yet many practitioners engaged in midwifery have passed through a long life without having met with more than two or three instances of this kind. Dr. Willan states that a physician of eminence, in attending 2982 ladies, during a period of 24 years, only lost one patient by laceration of the uterus—and our author thinks, from his own conversations and enquiries, that this may be looked upon as a fair average among females in the better ranks of life. This, however, can be little more than a mere guess.

"Should further observations tend to support this conjecture, it will, I conceive, admit of explanation on the following principles.—In the first place, females in the lower walks of life are, from their occupations, habits, &c. much more exposed to falls, bruises, and other accidental injuries during pregnancy, in consequence of which, the uterus may be either ruptured at the time they have sustained the violence, or may be so weakened in structure at some particular point, as readily to give way during its efforts to accomplish delivery. Secondly, from inattention during infancy and childhood; from poor, spare diet; from confinement in a close, impure atmosphere; and a variety of causes, they are more subject to Rachitis, a disease which is well known in many instances to lay the foundation for a deformed condition of the bones which constitute the pelvis, thus proving a source of tedious and of difficult labours at an after period of life. Lastly, they are more liable to fall into the hands of ignorant, inexperienced midwives, who not unfrequently, with the view of expediting the process of delivery, rupture the membranes at an early period of the labour, in consequence of which, the firm unyielding head of the child is prematurely brought in contact with the passages, exciting by its pressure, swelling, inflammation, and an interrupted state of the circulation in the uterus and adjacent parts. In such a case should there, unfortunately, exist any disproportion between the parts of the mother, and the head of the infant, or should proper measures not be employed to obviate distressing symptoms, and that the labour pains continue to recur with extreme violence, there is great risk of the uterus giving way, the laceration being of course most likely to occur at that part where the greatest pressure has been sustained." 4.

The sex of the infant may have some influence. In the 20

cases above alluded to, there were 15 boys and five girls. Dr. Clarke (of Dublin) made the average girt of the male infant's head 14 inches, while that of the female, was only 13 inches and five-eighths. Although this may appear a trifling difference, and really is so, in natural labours, with well-formed pelvis, yet, in contrary circumstances, it may be of great importance. It is probably owing to this cause that a greater number of males than females come into the world still-born, and that, in the majority of cases requiring embryotomy, the infants will be found of the male sex.

*Symptoms of ruptured Uterus.* "When the uterus gives way spontaneously, that is in consequence of its own inordinate action, the following is, in general, the history of the case. The patient, who, in all probability, has had a succession of tedious and of difficult labours, aft[illegible] encountering for many hours, perhaps for days together, sufferings of the most acute and harassing description, and at a time when her anxious friends and attendants are impatiently looking forward to the happy moment which is to free her from her misery, and render her a joyful parent, is suddenly attacked with an agonizing, crampish pain, referable to some particular spot in the abdomen; during the intensity of which she exclaims, that something has given way within her; she becomes sick, vomits a little, and complains of the child having risen to her stomach. Her pains cease, or are altered in character; she looks pale and ghastly, her countenance expressing great mental and bodily distress; she writhes and twists, with the severity of her torture,* the hand being applied to that part which she describes as the principal seat of it; she sighs often; complains of stitches about the heart; gazes wildly and anxiously about her; has embarrassed breathing; and desires to be raised up in bed.

"When these symptoms are present the experienced practitioner will feel but little hesitation in deciding on the nature of the case; but if in addition, we find that there has been some hæmorrhage from the vagina, that the presenting part has receded, that the abdomen has become so exquisitely tender as to render the slightest pressure intolerable, having, at the same time, become more prominent, and of an irregular shape, and that some projecting part of the infant can be distinguished immediately under its parietes, the case no longer admits of the possibility of doubt." 8.

It is not to be expected indeed that, in every case of ruptured uterus, the symptoms will be so obvious as in the above portrait. If, for instance, the head of the child be impacted in the

"* Occasionally, however, the patient lies perfectly quiet on her back, with the knees drawn up; in this position the abdominal muscles are relaxed, by which the pressure on the lacerated part is, to a certain extent, diminished."

pelvis, and the rupture is confined to the muscular structure of the uterus, its peritoneal covering remaining entire, we shall be deprived of several of the leading marks. In the first place, there will be no discharge of blood, the vagina being blocked up—secondly, there will be no recession of the original presenting part—lastly, we shall be unable to distinguish, with accuracy, any part of the infant immediately under the abdominal parietes.

The uterine *pains* do not always cease when that organ becomes ruptured. In many cases they recur with tolerable regularity, at least, till the womb gets rid of its contents. In more than one instance our author has known the action of the uterus to return with sufficient force to effect the expulsion of the child *per vias naturales*.

"The first case in which I had an opportunity of witnessing this circumstance, occurred to me, about two years ago, in the neighbourhood of Sandymount. The patient had been in labour several days, under the care of a midwife, when I was first called to her; the arm of the child protruded as far nearly as the axilla, beyond the external parts, and the uterus was in close contact with the body of the child, the waters having been drained off for several hours. I determined on making a cautious attempt to turn the infant, and for this purpose introduced my hand slowly and gradually into the vagina; at the posterior and upper part of which I discovered a large transverse rent, that communicated freely with the cavity of the abdomen. Being satisfied that any further attempts to get at the feet would only endanger an increase of the laceration, I resolved on perforating the thorax; a measure to which I was the more inclined, as the arm bore evident marks of putrefaction. Accordingly, having freely evacuated the contents of this cavity, as also those of the abdomen, I fixed the crotchet over the lumbar vertebræ as low down as possible, and in this way readily succeeded in bringing the breech and lower extremities into the world. The poor woman having now become extremely exhausted, I gave her some wine, and allowed her to rest a little before proceeding to the remainder of the operation. After about twenty minutes, however, to my great surprise, the uterus began to act, and in a pain or two the entire body of the child was expelled; another pain followed, and with very trifling assistance indeed the entire process was completed. In a few hours after delivery she swelled up, was attacked with vomiting of a coffee-coloured fluid, and sunk rapidly. I was very anxious to have the body examined, but could not prevail on her friends to give their consent." 11.

The *shape* and *feel* of the abdomen are bad criteria of the accident under consideration, though, in connexion with other symptoms, they may assist in the diagnosis. Neither can the feeling of pain on pressure be altogether depended on. In some instances our author has known the patient make little or no complaint, when the hand was applied over the abdomen—besides, tenderness of the abdomen is no very unusual phenomenon, where there is no rupture of the uterus. The constitutional disturbance has been sometimes very trifling, for some hours after the accident—nay, even for some days, so as to excite considerable doubts as to the existence of rupture.

"A remarkable instance of this description I had an opportunity of witnessing, about two years since, at Mr. D————'s, Black-Rock. The patient, a florid, corpulent housekeeper, accustomed to live well, and exercise but little, had been in labour upwards of thirty hours, under the care of an experienced practitioner in the neighbourhood, when she was seized with several of the symptoms of ruptured uterus, such as retrocession of the presenting part of the child, cessation of the proper labour pains, hæmorrhage from the vagina, and great tenderness of the abdomen. I saw her in about two hours from the time of the accident, and found her lying quite tranquil on her left side across the bed, as if waiting for the return of pains. Her countenance was natural; her pulse but little disturbed; she had no vomiting; no difficulty of breathing; and, in short, was to all appearance so well, that it was with considerable difficulty I could prevail on the people about her, to permit me to make an examination. On introducing my hand, however, into the vagina, I found that the fœtus and secundines had escaped altogether among the bowels, through an extensive transverse rent in the cervix uteri, opposite to the projection of the sacrum. I turned the child with great facility, and experienced but little difficulty until I came to the head, which I was obliged to perforate behind the ear, in consequence of some deformity in the bones of the pelvis. I employed all the force I thought justifiable for the purpose of completing the delivery, but in vain; and I am satisfied that had I continued my extracting efforts much longer, I should have separated the trunk from the head. This poor woman, notwithstanding all she suffered during the operation, made little or no complaint, until the evening of the following day, when she was seized with vomiting of a dark-coloured fluid, pain, soreness, and swelling of the belly; her stomach rejected every thing; and she died in about forty hours from the time of the accident. She was the mother of six living children, with all of whom, I understand, she had severe protracted labours." 15.

On some occasions the uterus has been known to give way during the very pain that effected the delivery of the child—instances of which may be found in the works of Crantz and Guillimeau. Finally, it has happened (only one case is on record) where the patient has died with many of the symptoms of lacerated uterus, and yet, on dissection, only a few fissures were found on the peritoneal covering of the fundus uteri, the muscular parietes being entire. The only case of this kind which our author could find, is recorded by Dr. John Clarke, in the third volume of Transactions for the improvement of Medical and Surgical Knowledge.

Dr. M'Keever has been induced to detail at some length the symptoms of lacerated uterus, from a conviction that, in many instances, the little chance the patient has had of surviving the accident, was destroyed by the practitioner mistaking the true nature of the case. Thus, in the remarkable instance of Mrs. Wilkins, recorded by Mr. Goldson in his pamphlet on lacerations of the vagina, the child, from the cause stated, was allowed to remain nearly 24 hours pressing on the viscera, before assistance was given. And so of many other cases.

"Before concluding this part of my subject, it may not be superfluous to mention a circumstance which I have known, on more than one occasion, to excite a good deal of uneasiness in the mind of the practitioner, particularly in cases of arm presentation, where considerable force had been employed in order to bring down the feet of the child. I allude to a firm fleshy ring, with irregular jagged edges which may be felt on examining a patient a short time after the delivery of the infant and secundines, and giving very much the feel, particularly if we make a hasty examination, as if the uterus were lacerated. By passing the finger, however, round the entire circumference of this fleshy margin, provided no laceration has taken place, we will find it arrested by the angle of junction which it forms with the vagina. Whereas, should a rupture have really taken place, we will be able to feel either the naked surface of the peritonæum, or to pass our finger at once into the cavity of the abdomen. This fleshy ring is in fact nothing more than the mouth of the womb, which having dilated after the manner of a sphincter muscle, in order to allow the contents of the uterus to escape, is again beginning to form and resume its original dimensions." 20.

*Treatment of ruptured Uterus.* It is now pretty generally agreed that *immediate delivery* affords the best, if not the only chance of recovery in this dreadful accident. Yet the late Dr. Denman was among the foremost of its opponents. It is asserted indeed, that instances are on record to prove that a female may not only recover from the effects of ruptured uterus and escape of the fœtus into the general cavity of the abdomen, but that she may again become pregnant and have a favourable delivery, while discharging piecemeal, through various outlets, the putrid mass. If these statements be true (and many of them are more than doubtful) they only prove that Nature is capable occasionally of making the most astonishing efforts for the continuance of life. Such insulated cases, therefore, cannot serve as guides at the bedside, however they may inspire us with reverence for the wonderful resources of Nature.

To argue the propriety of immediate delivery in such melancholy cases, is now, our author thinks, unnecessary. The sensible and judicious remarks of Dr. Douglas on this subject having satisfactorily proved "that if the expedient of immediate delivery has failed to preserve life, it has, at all events, superseded no mode of practice which promised a chance of safety to the patient."

"There is one point, however, about which there still exists some difference of opinion even among the best informed and most experienced practitioners, I allude to those very unfortunate cases in which the uterus bursts, and the child escapes among the bowels, prior to the dilatation of the mouth of the womb and external parts. Should we, in such a case, persevere in our efforts to dilate the os uteri, and thus deliver by the natural passages, or would we afford the patient a better chance of surviving the accident, by cutting through the parietes of the abdomen, and extracting the infant by what has been termed, the spurious cæsarian section.—This is undoubtedly a point of much difficulty, and one that requires the most mature and serious deliberation; at the same time I may observe that the general impression amongst the most intelligent physicians and surgeons in this city, is decidedly in favour of the latter proposal. And indeed when we take into account the dangers attendant on a forcible dilatation of the passages, such a preference must at once appear both rational and just. 'To dilate the os uteri forcibly,' observes Mr. Burns,* 'and thus to extract the child, is a proposal so rash and so hazardous, that I know of none in the present day who would adopt it.' Instances have occurred, in fact, in which the whole body of the uterus has been torn from the vagina by violent attempts at dilatation. Such, it is likely, was the cause of death in many of the cases alluded to by Peu and Mauriceau, although these writers do not appear to have been altogether aware of the circumstance. The former, in his Pratique des Accouchemens, tells us 'that the patient must inevitably perish, if much force has been employed in the dilatation of the uterus:' and the latter, in his 49th Aphorism, informs us 'that in those cases of uterine hæmorrhage where it becomes necessary to hasten delivery for the purpose of saving the woman's life, and that on examination the os uteri is found *thick* and *rigid*, the patient will in general die; but that if we find it thin and dilatable, she will have a better chance of recovery.'—Now it is, I conceive, by no means improbable that in several of these cases the patients died, not from the mere loss of blood, but in consequence of the violence done to the uterus. It is obvious, from the rigid, unyielding state of the passages described by Mauriceau in this Aphorism, that there must have existed considerable contractile power in the uterus, a circumstance which would, in the majority of instances, have prevented the hæmorrhage proceeding to such an extent as to endanger the life of the patient." 26.

"* See Burns' Midwifery, p. 243."

Further, our author remarks, there are a few well authenticated cases on record, where the expedient of cutting into the abdomen has been attended with the most satisfactory results; whereas there is no instance whatever of recovery after a violent and forcible dilatation of the passages. Mons. Thebaud de Bois relates a case in which this operation was successfully performed several hours after the uterus gave way, although too late to save the infant. M. Lassus has also stated a case where this operation was twice performed on the same individual with complete success. As this case is very interesting, and bears directly on the subject under discussion, our author begs to introduce a few of the particulars.

"A young woman of robust constitution, and pregnant for the fourth time, was seized with labour pains at the full period of utero-gestation. One pain was so particularly severe as to occasion her to faint; immediately after which she had a discharge of blood from the vagina: the pains ceased, the faintings became more frequent, the extremities cold, and the pulse agitated. Convinced that the uterus was burst, and that delivery could not be effected in the natural way without endangering a further enlargement of the rupture, the operation of gastrotomy was performed eighteen hours after the accident, and in six weeks the woman was able to attend to her ordinary duties. This woman again became pregnant, and on the 30th December, 1779, Mons. Lambron was a second time called to her assistance. When he arrived he found her with slight pains, the os uteri being sufficiently dilated to enable him to ascertain that the head presented favourably. In this state she continued for some time, when she was seized with a sharp pain, and the unfortunate woman announced, by the shrillness of her cry, that the uterus was again burst. She fainted, and the pains became weaker. The head of the child receded, and could not be felt, even by carrying two fingers for some distance within the lips of the laceration. Gastrotomy was immediately had recourse to, and the child lived about half an hour. This operation terminated, as on a former occasion, in the complete recovery of the patient, who became pregnant for the third time, and was delivered naturally in August, 1781, of a healthy child, but of rather small size." 28.

In the Quarterly Journal of Foreign Medicine we also find a case related by Messrs. Latouche and Jopet, in which a similar operation was performed with favourable result, although the infant and secundines had been lodged for a considerable time among the bowels. The child, &c. were removed by the cæsarean operation, 12 hours after the rupture of the uterus, and the mother recovered.

When the head of the infant remains impacted in the pelvis after the laceration, there can then surely be but one opinion as to the propriety of speedy delivery, either by the forceps or perforator, according to the particular situation of the patient. Thus, if we are on the spot at the moment when the uterus gives way, and we can pass the finger freely round the head of the child, whose ears can be readily felt, then we might employ the forceps in order to save the infant's life if possible. In the majority of instances, however, the crotchet will be found the more eligible instrument—first, because there is generally some deformity of the pelvis impeding the application of the forceps—and secondly, because the infant very quickly dies after the accident, and therefore the additional pain of the forceps is to be avoided.

"With regard to the perforator, I have only to observe, that in order as much as possible to guard against the retrocession of the head, the opening in the cranium should be made, not in the most prominent point of that cavity, as in ordinary cases, but rather to one side; so that the force employed in perforating may be directed, not towards the axis, but rather against the walls of the pelvis. I recollect on one occasion, where, in my hurry to deliver the patient, I omitted attending to this circumstance, in consequence of which the child receded into the cavity of the abdomen, where I was obliged to follow it, and deliver by the feet; an operation which, independent of the enlargement which it must have occasioned in the rent, put the patient to considerably more pain and distress than she would otherwise have had to encounter." 31.

After delivery of the infant the placenta will generally be found lying detached in the vagina; and having removed it together with any loose clots of blood, we are next to examine whether any portion of intestine has become protruded through the rent, so that it may be returned. This is a very important point to be attended to. We are now to give the patient an opiate and a little wine, and direct her to be kept quiet. The after-treatment consists, for the most part, in endeavouring to allay vomiting by effervescing mixtures combined with small doses of laudanum, and to keep in check the inflammation which must necessarily succeed.

"And here I may observe, that on no account should we discontinue or relax our efforts, however deplorable the case may appear: our exertions in fact should cease only with the patient's life, there being now several satisfactory and well authenticated instances on record, where, notwithstanding the most unpromising and alarming appearances a complete and perfect recovery has taken place." 33.

Dr. M'Keever thinks that, should a female have the good fortune to survive the accident in question, and again become pregnant, the subsequent delivery should be accelerated by turning the child and bringing down the feet.

*Portending Symptoms.* It certainly would be extremely desirable to ascertain whether there are any symptoms that would lead us to suspect the approach of such a terrible accident as laceration of the uterus. Our author is satisfied that there are a few premonitory symptoms which occasionally present themselves, and on which some degree of reliance may be placed.

"Thus, if a patient who has had a succession of tedious and of difficult labours, bringing forth on some occasions dead children, and at another time requiring the use of the crotchet, should have, after the full dilatation of the passages and discharge of the waters, violent bearing down pains, with little or no intermission, constituting what the women term, not inaptly, *showers of pains*, without a corresponding advance of the infant;—if she complains that her labour is centered in one particular spot, as the sacrum or pubis;—if in the intervals of her pains she complains of a tight, crampish feel in the abdomen, accompanied with flushing of the face and great frequency of pulse; if she is very restless, and throws herself about the bed as if wild with agony, crying out that 'she is ready to burst, if something be not done for her,' we then have reason to dread this accident, and should act with the utmost vigilance and precaution. Under these circumstances we should, in the first instance, take a large bleeding from the arm, with the view of moderating vascular action and of promoting a relaxed state of the passages; after which an injection of starch and laudanum should be administered. But if, notwithstanding these measures, the pains still continue severe and unremitting, without any impression being made on the presenting part, artificial assistance, in some form or other, should not be long deferred. As to the particular kind of interference, much will of course depend on the particular circumstances of the case; at the same time we should carefully bear in mind, that destructive instruments are upon no account to be employed, unless sanctioned by the approbation and advice of a second practitioner.*" 36.

We must not, however, be frightened at the idea of a lacerated uterus in every case of laborious parturition, or we shall often make an unnecessary sacrifice of the infant's life, and expose the parent to great injury.

"If a patient in tedious labour is kept tranquil and cool, getting none but the lightest and mildest drinks;—if the bladder and rectum are attended to;—if we refrain from frequent examination, by which the parts are rendered hot and inflamed;—if we prevent her making premature efforts to bear down, by which her strength and spirits are exhausted;—but above all, if we avoid an early discharge of the waters, it is truly astonishing the length of time a woman will bear even the

* This, of course, cannot apply to practitioners in the country, who are often so remote from professional aid as to render consultation impossible.

most vigorous and unremitting action of the uterus.* Frequently have I seen females forty, fifty, nay sixty hours, or more, in severe and almost uninterrupted labour, during which time they have taken no sustenance whatever, except a little tea and plain gruel, with perhaps a small allowance of bread; yet, after all, by an attention to the circumstances already enumerated, have brought forth living children, and have recovered without a single bad or even unpleasant symptom." 38.

---

*Cases of Recovery.* These are few in number. Still there are some recorded by Douglas, Hamilton, Clarke, Labatt, Frizelle, and Power. Dr. M'Keever adds two cases from his own observations, the first of which is so interesting, that we shall give a pretty full abstract of it in this place.

*Case.* A robust young woman was taken in labour in the evening of July 29th, 1820; and, after 30 hours' severe and unremitting labour, with great pain and tenderness of abdomen, she was seized with vomiting, and tendency to syncope. The midwife, becoming alarmed, sent for a surgeon, who found the patient apparently in a dying state; but, with the view of giving her a chance, he perforated the head of the child, which was firmly impacted in the first passages. The delivery was terminated by the crotchet. The placenta was next removed with great difficulty, and, on introducing his hand into the vagina, the accoucheur discovered a rent high up at its posterior part, extending round to the neck of the bladder, and communicating freely with that viscus. Some wine and laudanum were given to the patient, and she recruited considerably. The vomiting was relieved, and she got some sleep. The following day she made little complaint. In the afternoon, however, one of the attendants observed a shining substance hanging from the external parts, but did not do any thing except passing a piece of linen through the loop. *August 1st.* Made no particular complaint. *August 2nd.* One of the attendants, in attempting to remove the protruding substance, gave the patient great pain, and, from this moment, a train of the most formidable symptoms set in. The abdomen swelled up and became very painful—she had incessant vomiting, and complained of much dragging, lacerating pain in both iliac regions. In this state she continued, with little variation of symptoms,

"* 'Since it became usual to keep women in labour in a cool atmosphere, to prevent them making voluntary exertions during the dilatation of the os tincæ, and to support them by mild, instead of stimulating nourishment, the powers of the constitution fail but seldom in expelling the fœtus, when there is no material defect in the formation of the pelvis.'—*Dr. Clarke's Report of the Dublin Lying-in-Hospital.*"

till Friday, the 5th August, when our author saw her for the first time.

"It would be difficult to conceive a more melancholy or distressing picture of human misery than she at this time presented: her belly was much swoln, and excessively painful; her stomach rejected even the mildest articles of diet; the bowels were still obstinately confined; she had a small, intermitting, and tremulous pulse, and her countenance was pallid and ghastly: in short, she had every appearance as if a few hours at furthest would put a period to her sufferings. On raising the bed-clothes for the purpose of inquiring into the precise state of matters, I found, in place of the alleged portion of membrane, near a yard and a half of her bowels coiled up under her, black, and to all appearance putrid, while the cylinder of the intestine was in many parts so incomplete, that the finger could be passed freely up and down through the rents.

"In this very melancholy state of affairs, it is hardly necessary to say that my prognosis of this poor woman's case was of the most alarming nature. To have attempted any thing in the way of operation, under the present circumstances, would, I conceived, be not only impracticable, (owing to the exquisitely tender state of the passages,) but absolutely injurious, by destroying the only chance which I conceived she had of prolonged existence, that of having an artificial anus established in the Vagina. In fact, it appeared to me that, except in the way of palliating distressing symptoms, little or nothing could be done for her; and in this view of her case I was confirmed by the superior judgment of Dr. Labatt, at that time Master of the Lying-in-Hospital. The common saline mixture, with small doses of tincture of opium, was accordingly ordered, with the view of relieving the extremely irritable state of her stomach, and she was directed to take some *cold* chicken broth for her ordinary drink.* A pill, consisting of three grains of calomel and half a grain of opium, was directed to be taken every four hours; the abdomen was ordered to be well stuped, and she was allowed a little wine occasionally. On the following day I found the abdomen still greatly swelled, but thought it bore pressure with somewhat greater freedom; the vomiting and hiccup continued incessant; bowels obstinately confined; the extremities were cold; the face collapsed, and covered with a cold sweat: The protruded portion of intestine had a soft, doughy feel, was more shrivelled, and, instead of being black and livid, as when I first saw it, was of a dirty ash-colour. The remedies already mentioned were directed to be continued, and in addition she was allowed, at her own earnest desire, a table-spoonful of barm occasionally.

"August 7th. As I was in daily, I might say hourly expectation of hearing of this poor woman's dissolution, it afforded me no small gratification and surprise to find, on visiting her this day, that the mor-

"* I have frequently known *cold* chicken broth remain on the stomach when every other article of diet has been rejected."

tified portion of intestine had come away in the course of the preceding night, and that she had since been nearly altogether free from alarming and distressing symptoms.* The vomiting and hiccup had ceased, her pulse was regular and of good strength, her countenance was much improved, and the abdomen less tender, though still much swelled. About an hour after the sphacelated piece of intestine became detached, she had a copious discharge of fæces by the Vagina, being the first alvine evacuation she had since delivery. The pills and cold chicken broth were directed to be continued, and her attendants were instructed to syringe the Vagina occasionally with a warm decoction of camomile flowers.

"Friday, August 11th. Has been mending daily since last report: the tension and soreness of the abdomen are still further diminished, and the mammæ are now distended with milk; pulse natural; tongue clean; expresses a desire for food. As the fæces and urine were both passed involuntarily per Vaginam, she was directed to wear a T bandage, with a large soft sponge to the external parts, for the purpose of absorbing the discharges: a pint of porter, with a small quantity of wine, were allowed daily; and a mild, nourishing diet, consisting for the most part of eggs, beef tea, and milk, was recommended." 48.

From this time she mended. Her countenance improved—the secretion of milk became abundant—the excrementitious matter voided, per vaginam, was of a light yellow colour, and free from fæcal smell. In the course of a few weeks she was removed to the Lying-in-Hospital, where she was examined by several eminent medical gentlemen.

It is now more than three years since the accident occurred, and since some account of it was laid before the public, in the second volume of the Transactions of the Medical Association.

Her general health is much improved. She can walk a dozen of miles without inconvenience. She has become *fat*—a circumstance that upsets Sir Everard Home's theory, of fat being formed in the large intestines, for here the colon is necessarily in a state of complete inactivity. For two years after her confinement she had no discharge whatever from the rectum, the residue of her food being altogether voided by the vagina. About the end of that period, however, she was attacked with violent bearing-down pains, accompanied by tenesmus, and, after half an hour's severe sufferings, she discharged, by the natural passage, a large quantity of dark, pitchy-co-

"* It measures precisely three feet eleven inches, and is at present in my possession: the members of the Medical Association of this city had an opportunity of inspecting it when I first gave notice of this remarkable case. There were present on that occasion, Dr. Clarke, senr. Dr. Clarke, junr. Doctor Crampton, Dr. Teeling, with several other leading members of the profession."

loured fæces, of the consistence of balls of firm wax. The stools have since been discharged by both passages, and the quantity, per vaginam, has been gradually diminishing during the last four or five months—and for six weeks past they have been entirely voided *per vias naturales.* It is to be stated, also, that she became pregnant lately, and that the return of the fæces to their natural course was in proportion to the increasing size of the uterus. She was delivered safely of a small child, and nothing particular occurred in parturition. The fæces passed the natural way, and our author, on an examination, could discover no trace of the artificial anus. Unfortunately she has never recovered the power of retaining her urine, and is, therefore obliged to wear a T bandage, with wadding of fine linen, to obviate the misfortune.

From a review of the foregoing remarkable history, our author comes to the following conclusions:

"First. That a case of lacerated vagina, even though it be accompanied with a rupture of the bladder, together with the loss of a considerable portion of intestine, cannot be considered as a necessarily fatal occurrence.

"Second. That life may be prolonged, and the system be properly nourished, notwithstanding the loss of nearly four feet of the *small* intestine.*

"Third. That in such a case, the loss of absorbent surface is compensated for by an increased demand for food, as also by the patient selecting those articles of diet which afford most nutriment in a given bulk.

"Fourth. That, notwithstanding the increased desire for food, less nutriment is taken at any one time; a kind of continued sympathy appearing to be established between the stomach and small intestine, in consequence of which the supply is proportioned to the diminished extent of surface afforded for absorption.†

"Fifth. That aliments pass from the stomach, not in the order of their admission into that cavity, but according to their greater or lesser nutribility; those substances that afford least nutrition passing first, while those which afford most are retained a longer time.

"Sixth. That fat may be deposited in considerable quantity under the integuments of the body, although the large intestine, which

"* I have, within these few days, submitted this portion of intestine to Mr. John Shekleton, Demonstrator of Anatomy to the Royal College of Surgeons; and in a written communication with which I have been favoured by that gentleman, he states that, after a careful and attentive examination, he has no doubt whatever of its being *small* intestine; and that, from the entire absence of valvulæ conniventes and villi on its mucous surface, he considers it to be a portion of the ileum, near to its termination."

"† Nutritive substances are also found to be more readily absorbed when taken in small quantities at a time, than when presented in more considerable quantities, as is exemplified in the universally good condition of cooks, and their attendants.—*See Paris's Pharmacologia*, p. 153."

has been supposed to be the laboratory where this substance is formed, has been in a state of complete inactivity.

"Seventh. From the length of time the fæces were retained in the large intestine, it affords a striking and convincing proof that they do not consist, as has been supposed, of the residue of our food in a state approaching to putrefaction; but that so long as they are retained in the body, the vital changes which they undergo preserve them from decomposition.

"Eighth. That an artificial anus, not the consequence of a strangulated hernia, may become completely obliterated, and the fæces resume their natural healthy track.

"Ninth. That the changes naturally consequent on gestation and parturition, so far from interfering with the process of obliteration, appear rather to accelerate its progress." 61.

*Case* 2. The second successful case is very short, and the history may be given in a few words. Mary Kelly, aged 20, was admitted into the Lying-in Hospital, on the 11th of September 1821, in labour of her second child. Her pains continued incessant and severe till six o'clock the following morning, when they suddenly ceased, and she had some hæmorrhage from the vagina. The head receded, and, on pressing over the pubis, the child could be distinctly traced through the abdominal parietes. Her countenance was pale and ghastly, with a peculiar death-like expression, and she had some vomiting. The head was perforated, and the delivery accomplished with considerable difficulty, owing to some deformity in the bones of the pelvis. A large gush of blood followed the extraction of the placenta, and on introducing the hand into the vagina, a considerable rent was discovered at the anterior part of the cervix uteri, through which the abdominal viscera could be distinctly felt. The body of the uterus was found firmly contracted, like a large ball, at the back part of the pelvis. After delivery, she had some wine and an opiate. Her pulse was low and weak, but she had no return of vomiting, and complained of pain only in the region of the uterus. Some symptoms of inflammation followed, and were combated by the proper antiphlogistic means. She mended progressively, and was ultimately discharged from the Hospital cured. Three months afterwards she returned, at Dr. Labatt's desire, and reported herself quite well.

A number of other cases are detailed; but as they were all fatal, we shall pass them over, referring to the original those who wish to consult them. We return thanks to Dr. M'Keever for the pleasure and information afforded by his little work, of which we have laid a copious analysis before our readers.

